# Supplementary material for: Identification of photoperiod-induced specific miRNAs in the adrenal glands of Sunite sheep (Ovis aries)
Source: Front Vet Sci. 2022 Jul 22;9:888207. doi: 10.3389/fvets.2022.888207 (PMC9354845; doi:10.3389/fvets.2022.888207)
Supplement: Supplementary file 1 [file Data_Sheet_1.ZIP › Supplementary file/miRDeep2 file.zip!/miRDeep2 file/Know_Structure/LP42A1/oar-mir-10a.pdf]

miRBase precursor : oar-mir-10a
Total read count : 159183
oar-miR-10a read count : 158346
remaining reads : 837

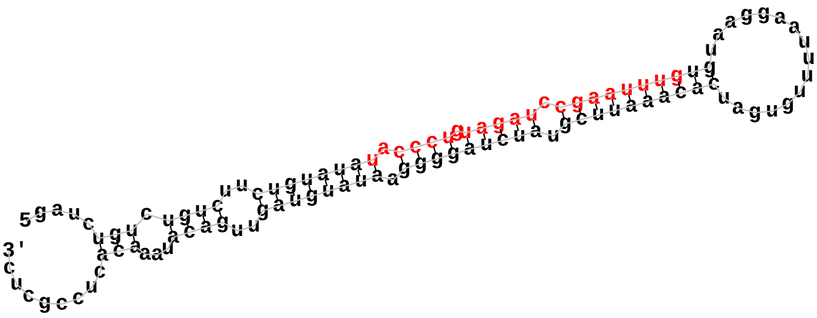

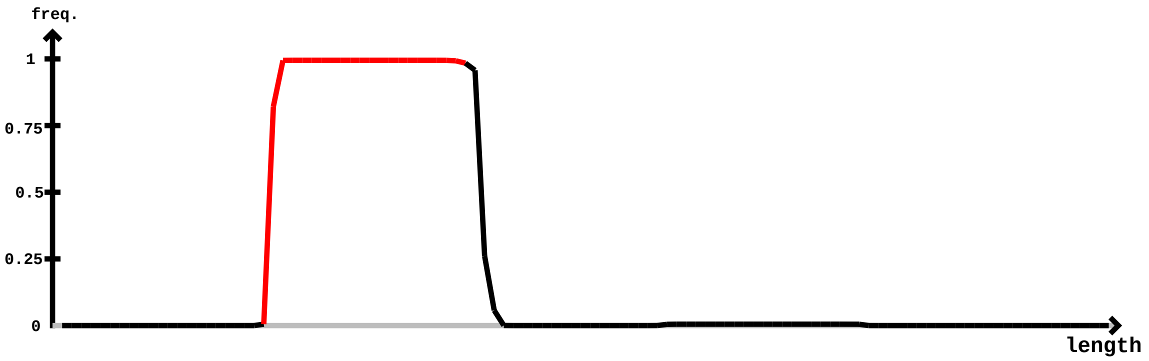

oar-miR-10a

```
5'- gaucugucugucuucuguauauacccuguagauccgaauuuguguaaggaauuuugugaucacaaauucguaucuaggggaauauguaguugacauaaacacuccgcuc   -3'    exp
    ....(((.((((..((((((((.((((.(((((.((((((((((................)))))))))).))))))))).))))))))..))))...)))........    reads      mm         sample
    ..ucugucugucuucuguaua........................................................................................    2          0          seq
    ....................auacccuguagauccgaa.......................................................................    1          0          seq
    ....................auacccuguagauccgaau......................................................................    1          0          seq
    ....................auacccuguagauccgaauu.....................................................................    5          0          seq
    ....................auacccuguagauccgaauuu....................................................................    63         0          seq
    ....................auacccuguagauccgaauuug...................................................................    126        0          seq
    ....................auacccuguagauccgaauuugu..................................................................    593        0          seq
    ....................auacccuguagauccgaauuugug.................................................................    47         0          seq
    ....................auacccuguagauccgaauuugugu................................................................    9          0          seq
    .....................uacccuguagauccgaa.......................................................................    1          0          seq
    .....................uacccuguagauccgaau......................................................................    29         0          seq
    .....................uacccuguagauccgaauu.....................................................................    241        0          seq
    .....................uacccuguagauccgaauuu....................................................................    1148       0          seq
    .....................uacccuguagauccgaauuug...................................................................    3601       0          seq
    .....................uacccuguagauccgaauuugu..................................................................    97412      0          seq
    .....................uacccuguagauccgaauuugug.................................................................    22388      0          seq
    .....................uacccuguagauccgaauuugugu................................................................    5060       0          seq
    .....................uacccuguagauccgaauuugugua...............................................................    20         0          seq
    .....................uacccuguagauccgaauuuguguaa..............................................................    3          0          seq
    ......................acccuguagauccgaau......................................................................    3          0          seq
    ......................acccuguagauccgaauu.....................................................................    43         0          seq
    ......................acccuguagauccgaauuu....................................................................    167        0          seq
    ......................acccuguagauccgaauuug...................................................................    537        0          seq
    ......................acccuguagauccgaauuugu..................................................................    12800      0          seq
    ......................acccuguagauccgaauuugug.................................................................    10018      0          seq
    ......................acccuguagauccgaauuugugu................................................................    3933       0          seq
    ......................acccuguagauccgaauuugugua...............................................................    19         0          seq
    ......................acccuguagauccgaauuuguguaa..............................................................    1          0          seq
    .......................cccuguagauccgaauuugu..................................................................    20         0          seq
    .......................cccuguagauccgaauuugug.................................................................    34         0          seq
    .......................cccuguagauccgaauuugugu................................................................    17         0          seq
    ........................ccuguagauccgaauuugu..................................................................    2          0          seq
    ........................ccuguagauccgaauuugugu................................................................    1          0          seq
    ........................ccuguagauccgaauuugugua...............................................................    1          0          seq
    .........................cuguagauccgaauuugu..................................................................    2          0          seq
```

oar-miR-10a

```
gaucugucugucuucuguauauacccuguagauccgaauuuguguaaggaauuuugugaucacaaauucguaucuaggggaauauguaguugacauaaacacuccgcuc
...........................................................ucacaaauucguaucuagggg.............................    1      0      seq
...........................................................ucacaaauucguaucuaggggaa...........................    1      0      seq
............................................................cacaaauucguaucuaggggaau..........................    2      0      seq
.............................................................acaaauucguaucuaggggaau..........................    27     0      seq
..............................................................caaauucguaucuagggg.............................    1      0      seq
..............................................................caaauucguaucuagggga............................    12     0      seq
..............................................................caaauucguaucuaggggaa...........................    19     0      seq
..............................................................caaauucguaucuaggggaau..........................    677    0      seq
..............................................................caaauucguaucuaggggaaua.........................    6      0      seq
..............................................................caaauucguaucuaggggaauau........................    3      0      seq
...............................................................aaauucguaucuagggga............................    3      0      seq
...............................................................aaauucguaucuaggggaa...........................    1      0      seq
...............................................................aaauucguaucuaggggaau..........................    70     0      seq
...............................................................aaauucguaucuaggggaaua.........................    7      0      seq
...............................................................aaauucguaucuaggggaauau........................    1      0      seq
................................................................aauucguaucuaggggaau..........................    1      0      seq
................................................................aauucguaucuaggggaauau........................    3      0      seq
```
